# Training load and intensity in triathlon: objective differences between sex, age, race distance preference and training phase across a cohort of 95 age-group triathletes over six months

**DOI:** 10.3389/fspor.2026.1798702

**Published:** 2026-04-30

**Authors:** Leighton A. Wells, Samantha M. Hoffmann, Lyndell Bruce, Peter Kremer, Dan B. Dwyer

**Affiliations:** 1 Institute for Physical Activity and Nutrition (IPAN), Centre for Sport Research (CSR), School of Exercise and Nutrition Sciences (SENS), Deakin University, Melbourne, VIC, Australia; 2 School of Exercise and Nutrition Sciences (SENS), Deakin University, Melbourne, VIC, Australia

**Keywords:** average distance, average volume, training stress score, TrainingPeaks, TSS, triathlon coaching, training stress score

## Abstract

**Aim:**

This retrospective cohort study aimed to describe a large dataset of objective training load (TL) data from training sessions of age-group triathletes and to compare sex, age group, race distance preferences, and training phases.

**Methods:**

An online survey collected TL data from 95 age-group triathletes (18 female, 77 male) in 25 countries. The data included the distance, duration, average heart rate, and training stress score (TSS) for 34,731 training sessions. A Linear Mixed Model analysis was completed, and neither age nor sex was associated with TL (*p* > 0.05), and mean heart rate (% of HRmax) was not associated with any factors.

**Results:**

The TL of long-course triathletes (615 min./wk, 171 Km/wk, 574 TSS/wk) was higher than that of short-course athletes (507 min./wk, 118 Km/wk, 452 TSS/wk). There were differences (*p* < 0.05) in TL between four training phases, with the ’specific’ phase having the highest TL (556 min./wk, 166 Km/wk, 620 TSS/wk) and off-season the lowest (340 min./wk, 84 Km/wk, 364 TSS/wk). Differences (*p* < 0.05) in TL based on race distance and training phase align with evidence-based practice.

**Conclusions:**

Comparisons of TL with the cohort values reported here may be helpful to coaches and athletes, given the previous absence of objective age-group athlete datasets.

## Introduction

Triathlon, with its varying distances from sprint to Iron-distance races, places different training demands on the athlete. The ability to prescribe and manage appropriate training loads and allow for adequate athlete recovery across these disciplines is paramount for optimising performance and preventing injury (1). Training load (TL) encompasses various internal and external load metrics, such as heart rate (HR) (2), distance (kilometres or miles) (3), duration (hours) (4), power output (watts) (5), Training Stress Score (TSS) (6, 7), Rate of Perceived Exertion (RPE) (8), and the subjective feedback of athletes (9, 10). Collectively, they provide a comprehensive overview of an athlete's training status (11) and their physiological and psychological responses (12, 13).

Technological advancements in sports science have led to a paradigm shift in how training data is collected and analysed (14, 15). Modern training management systems (TMS) like TrainingPeaks® (14) offer platforms for data collection that include objective metrics such as speed, power, cadence, stroke rate, laps, HR, pace, and distance. TL data is uploaded to a TMS from training devices, such as GPS sports watches (16) and power meters (17, 18), thereby removing the need for manual recording and providing a better opportunity to understand and manage TL (19). Despite these advancements and Etxebarria's guidelines (1) that emphasise the importance of managing triathlon TL, this type of data has not been used to describe how TL might vary by sex, age, race distance and training phase.

The TL of triathletes, such as weekly training duration and distances for each discipline, has been reported previously. Studies by Sinisgalli et al. (3), Gulbin et al. (20) and Ansell et al. (21), reported the TL of Ironman and ultra-endurance triathletes but not short-course triathletes. More recently, Falk-Neto et al. (22) reported the TL of nine Olympic-distance triathletes, using a variety of TL variables, focusing on the six weeks before and two weeks after a race. Most recently, Vleck et al. (23) provided an expansive report and comparison of the training load of 48 amateur short-course triathletes. This study was uniquely valuable because it included measures of training intensity and frequency, differences in TL between training phases, and measures of “life stress”.

A key aspect of the value of TL data is knowing whether and how it varies by sex, age, race-distance and training phase. This allows coaches and triathletes to compare their own TL with data from relevant research cohorts (e.g., their age, race, and distance preference), helping them understand whether their TL is comparable. The triathlon TL studies mentioned in this introduction do report TL for different ages, race distances and phases. However, understanding differences in TL between race distance preferences requires comparisons across studies conducted in different eras (i.e., 1999, 2012, & 2021–2023) that used different data collection and analysis methods, which is problematic. The validity of the reported TL varies, as some studies were based on 9 triathletes (22) and others on 99 triathletes (3). Furthermore, the period over which TL was quantified ranged from one week (3) to one year (23), and although several studies included both males and females, none compared TL between sexes. Finally, the TL data reported across all these studies relied entirely on athletes’ memory and estimates of their previous TL, both of which are susceptible to error and bias (24). Borresen et al., when comparing self-reported (subjective) training duration to device-recorded (objective) training data (via GPS sports watch), found that up to 41% of training data was under- or overestimated (4). The TL derived from self-report questionnaires may not be as accurate as TL data obtained from long-term objective records. There are no reports of objective-based triathlon TL data (i.e., data uploaded to a TMS from training devices) in the literature, highlighting a critical gap.

TSS is highlighted as a key metric for assessing TL, but there are no age-group triathlete training datasets in the literature that use it. TSS is a TL metric that combines exercise intensity and duration, making it a valuable tool for evaluating TL across disciplines. Using Banister's training impulse (TRIMP) concept, TSS integrates session duration, mean HR (or power or pace), and intensity into a single measure. The validity of TSS and its agreement with TRIMP have been demonstrated for cycling, running, and HR-based assessments (12, 25, 26). In the present study, TSS is utilised as a unified TL metric encompassing data from swimming, biking, running, and HR measures. *(Note: TSS and its associated calculations used by the TMS are explained in further detail later in the Methods section.)*

Detailed triathlon TL data, including training phases, race distance preferences, and age groups, could assist coaches and athletes in providing meaningful data from a relevant cohort to compare their training. However, large-scale datasets that enable robust normative TL values in triathlon are difficult to obtain. In the absence of established normative load values, providing a strong, objective indication of what triathlon training for age-group athletes may look like is a meaningful step forward. Therefore, this study aimed to describe objective loads for these categories in a self-selected cohort of age-group triathletes who use TrainingPeaks by examining TL metrics (duration, distance, HR, TSS) and strength-training data.

## Methods

### Participants

One hundred and two age-group triathletes were recruited for the study, with ninety-five (77 male, 18 female; age 48.0 ± 12.2 years) providing usable data. Participants were recruited directly through various online channels, including social media platforms [Twitter (X), LinkedIn, Facebook, and Instagram], triathlon website forums, direct emails to triathlon clubs, and targeted communications to the TrainingPeaks customer bases of both coaches and athletes via TrainingPeaks’ global e-newsletters. The breadth of the sample aimed to capture a wide range of training habits and strategies employed across different geographies, age groups, and race-distance preferences. Athletes were selected based on having at least 12 months of triathlon training experience, using TrainingPeaks® (Peaksware, Boulder, CO, USA) as their primary TMS, and having at least 6 months of continuous training data with minimal breaks. Before participating, a consent statement informed participants that the institution's ethics committee had approved the study. By proceeding to upload their training data and complete the associated survey, participants provided informed consent for their data to be collected. Given this recruitment strategy, the sample should be considered a self-selected convenience cohort of TrainingPeaks users rather than a representative cross-section of all triathletes across age groups.

### Design

This retrospective cohort study was designed to analyse six months of training data extracted from the TrainingPeaks TMS by age-group triathletes. The design allowed for descriptive comparisons of TL across multiple factors, including training phase, race distance preference, and age-group categories (21, 22). As part of this analysis, metrics across the modalities of swim, bike, run, and strength training were analysed. These metrics included distance, duration, average HR, TSS, and RPE over a recent six-month training period.

### Methodology

Participants were asked to provide historical training data spanning six months, exported from their TrainingPeaks account. Guidance on how to complete the export was provided in a pre-recorded, narrated instructional video to ensure data export consistency and maintain participants’ anonymity. After uploading their training data, the second part of the data collection process involved athletes answering survey questions about their age-group race category, birth sex, race-distance preference, training phases related to the uploaded data, and their strength-training habits. The data and responses to survey questions were collected using an online survey system (Qualtrics, Provo, UT, USA). No textual comments or personal annotations were analysed in the datasets, thereby maintaining privacy and avoiding the extraction of personally identifiable information.

The primary load metric selected was TSS because it quantifies both exercise intensity and duration. It is based on objective data directly uploaded from training devices and is therefore not subject to recall bias (24). In this study, TSS and its swimming, cycling, and running derivatives (calculations as shown in Table 1) are explicitly treated as TL dose indices (i.e., the amount of TL completed) rather than stress-response measures (e.g., RHR, HRV, RPE, mood). Acute physiological and psychological stress responses to load were not measured. This aligns with the study objective (quantifying TL exposure) and relates to the recent work of Impellizzeri et al. (27). It is acknowledged that different intensity–duration combinations may not be equivalent in their downstream stress-response effects; our analyses, therefore, interpret TSS as TL dose (27), with stress-response considerations outside the scope of this study. It is also noted here that TSS data depends on accurately entered biometrics, thresholds, device calibration, and data validity across disciplines. This is particularly relevant in recreational athletes and in swimming, where validation is more limited.

**Table 1:** Training stress score (TSS) calculations for various sports modalities.

Version of Training Stress Score (TSS)	Description
sTSS	Swim Training Stress Score*: [(NSS/FTSP)^3^] × Duration (in hours) × 100 = the average swim speed for the session, and FTSP = the threshold pace determined from a time trial. * NOTE: sTSS, while not currently validated, is based on validated methods of calculating load.
TSS	Power-based Training Stress Score—Cycling/Running (7, 25): (sec × NP × IF)/(FTP×3,600) × 100 = duration of the workout in seconds, NP = Normalised Power (adjusted power reflecting intensity variability), IF = Intensity Factor [ratio of NP to Functional Threshold Power (FTP)], FTP = the highest power sustainable for approximately one hour.
rTSS	Run Training Stress Score (12): (sec × NGP × IF)/(FTP × 3,600) × 100 = duration of the workout in seconds, NGP (Normalised Graded Pace) adjusts pace for changes in terrain, IF = the Intensity Factor (NGP/FTP), FTP = the Functional Threshold Pace for running.
hrTSS	Heart Rate-Based Training Stress Score) (26): Similar to Edward's TRIMP (eTRIMP) (25), hrTSS is estimated based on time spent in each heart rate zone relative to the athlete's threshold heart rate. This measure is suitable for workouts lacking power or pace data.

TSS calculations are based on the individual's unique capacity. TSS normalises load across athletes, so, irrespective of whether they are beginner or advanced-level age-group athletes, younger or older, male or female, TSS is a relative load measure based on their unique training thresholds.

TSS load values do not reflect an absolute difference; for example, an athlete with a 150-watt Functional Threshold Power (FTP) (5) vs. a 300-watt FTP will not have different load values for the same combination of intensity and session duration. Intensity Factor (IF) (28) in the TSS calculations serves to convert the athlete's work rate into a percentage of their unique pace, power, and heart rate thresholds.

TSS and related metrics were calculated within TrainingPeaks, based on the following formulae (Table 1):

### Data processing and analysis

All data processing was completed using the R programming language (RStudio, PBC, Boston, MA, USA). Initial data preparation involved data removal to ensure data integrity. This included the removal of implausible TL values; 1) Swim – TSS <7.5 and >200, sessions greater than 2 h, 2) Bike—training heart rates below 70 bpm, workout durations >7 h, TSS >550, distance <3 km, 3) Run—workout >4 h, TSS >400, and 4) non-triathlon activities and duplicate records. We determined these thresholds based on our experience with mistakes that can occur when recording training sessions with technology (e.g., recording a session that was started but abandoned quickly, and forgetting to stop recording after a training session has finished). The raw data contained 35,229 training sessions, which were reduced to the number of sessions and individual training weeks reported in the Results. Sessions classified as ‘race’ within TrainingPeaks were retained and contributed to the weekly training-load totals, primarily within the Taper/Race/Post phase. It is noted that factors such as the athlete's speed in training have not been included due to variability in environmental conditions and terrain across a globally diverse cohort.

The traditional triathlon age-group race categories (20–24, 25–29, 30–34, through to 65+) could not be used for age-group descriptive analysis due to insufficient participant numbers in some age categories (less than 12 per group), thereby impacting statistical power. Therefore, age groups were adjusted to align more closely with race categories. For example, some categories in the present study, such as 18–34 and 59+, span 16 years, compared with the traditional 5-year intervals (29). Training phases were categorised into general/base, specific/build, and off-season, with the shorter phases of taper, race, and post-race grouped together due to their potential to occur within a month. Although these terms are common to describe training phases, descriptions of each phase were provided in the athlete survey to avoid misinterpretation. Comparisons were completed in R using several packages (Tidyverse 2.0.0, lme4 1.3–38, lmerTest 3.2-0, performance 0.15.3, effectsize 1.0.1) (30).

To evaluate associations between the four factors (independent variables: sex, age group, race-distance preference, training phase) and the training outcomes, a Linear Mixed-effects Model (LMM) was fitted separately for each dependent variable: total weekly duration, total weekly distance, total weekly TSS, and mean weekly HR. The design was unbalanced (i.e., not all athletes contributed data to every combination of factor levels across phases), so the LMM was used to estimate factor effects under these unequal cell sizes. Athlete ID was included as a random factor to account for repeated weekly observations within athletes. The structure of the model was; training load variable∼sex + age grou*p* + race distance category + training phase + sex * race distance category + sex * training phase + (1| athlete id). The LMM results [F-statistics, *p*-values, Cohen's f (partial) effect size with 95% confidence interval] are reported for each dependent variable (Table 2). Where a factor showed a significant effect (*p* < 0.05), Bonferroni-adjusted pairwise comparisons were used to identify which levels differed. In addition to inferential outputs (LMM and Bonferroni), descriptive summaries (means and/or percentiles) are reported in the Results and Supplementary Tables for cohort benchmarking.

**Table 2:** Factors examined in relation to training load.

	Load measure/Factor in the linear mixed model				
		Total duration per week (hrs)	Total distance per week (m)	Average session HR per week (bpm)	Total load per week (TSS)
Model fit (LRT *X*^2^, *p* value)		105.3, *p* < 0.001	59.8, *p* < 0.001	16.4, *p* = 0.45	112.5, *p* < 0.001
Factor	df	F statistic, *p* value, Cohen's f (partial) and 95% CI			
Sex	1	F = 0.14, *p* = 0.70, f_p_ = 0.04 (0.00–∞)	F = 0.001, *p* = 0.97, f_p_ = 0.003 (0.00–∞)	F = 0.02, *p* = 0.88, f_p_ = 0.01 (0.00–∞)	F = 0.06, *p* = 0.81, f_p_ = 0.04 (0.00–∞)
Age group	5	F = 0.08, *p* = 0.99, f_p_ = 0.07 (0.00–∞)	F = 0.52, *p* = 0.76, f_p_ = 0.17 (0.00–∞)	F = 1.24, *p* = 0.29, f_p_ = 0.26 (0.00–∞)	F = 0.32, *p* = 0.89, f_p_ = 0.20 (0.00–∞)
Race distance preference	2	F = 5.20, ***p*** **=** **0.007**, f_p_ = 0.33 (0.13–∞)	F = 3.54, ***p*** **=** **0.03**, f_p_ = 0.27 (0.06–∞)	F = 1.87, *p* = 0.16, f_p_ = 0.20 (0.00–∞)	F = 4.62, ***p*** **=** **0.01**, f_p_ = 0.28 (0.06–∞)
Training phase	3	F = 13.1, ***p*** **<** **0.001**, f_p_ = 0.14 (0.10–∞)	F = 12.49, ***p*** **<** **0.001**, f_p_ = 0.13 (0.09–∞)	F = 0.46, *p* = 0.70, f_p_ = 0.03 (0.00–∞)	F = 20.5, ***p*** **<** **0.001**, f_p_ = 0.08 (0.06–∞)
Sex × Race distance preference	2	F = 0.71, *p* = 0.49, f_p_ = 0.12 (0.00–∞)	F = 0.09, *p* = 0.91, f_p_ = 0.04 (0.00–∞)	F = 1.35, *p* = 0.26, f_p_ = 0.17 (0.00–∞)	F = 1.37, *p* = 0.25, f_p_ = 0.25 (0.00–∞)
Sex × Training phase	3	F = 5.1, ***p*** **=** **0.001**, f_p_ = 0.08 (0.01–∞)	F = 6.12, ***p*** **<** **0.001**, f_p_ = 0.09 (0.05–∞)	F = 0.92, *p* = 0.42, f_p_ = 0.04 (0.00–∞)	F = 2.54, *p* = 0.054,f_p_ = 0.03 (0.00–∞)

A linear mixed model for each load variable was used to evaluate the effects of four factors. The goodness-of-fit of each model is reported as the likelihood ratio test (LRT Chi-squared) in comparison with a null model. The effect of each factor and two interactions in each model are reported as the F statistic, the associated *p*-value, Cohen's f (partial) as an effect size with a 95% confidence interval. “df” is degrees of freedom.

## Results

One hundred and two (102) individual athletes submitted the online survey, with 95 providing usable data for TL analysis. The final dataset comprised 34,731 individual training sessions, from which all four TL variables were extracted. Training load data were aggregated into calendar weeks, yielding 2,177 individual training weeks, an average of 22.9 per athlete.

### Demographics

The survey respondents ranged in age from 18 to 75 years (48.0 ± 12.2 years) and were predominantly male (male 81%, female 19%). Participants were primarily from Asia and Oceania (27.8%), North America (25.8%), and European countries (25.7%), but also from the UK (9.9%), Latin America (7.9%) and Africa/Middle East (3.0%). Approximately half of participants (52.5%) were coached, while half (47.5%) were not. Most respondents (69.3%) specialised in long-course events [Half-Iron Distance (70.3) and Iron-Distance Triathlons], and the remainder either specialised in short-course events (Sprint Distance and Olympic Distance; 11.9%) or did not specialise (18.8%).

### Factors affecting training load

The effect of four factors (i.e., sex, age, race distance and training phase) on each measure of TL was examined, and the modelling results are reported in Table 2. The model for each TL variable converged and performed well (i.e., significant model fit metrics). Athlete sex and age group did not have an effect on any TL variables. However, race distance preference and training phase were significantly associated with all TL measures except HR.

Average HR was not affected by any of the factors (*p* > 0.05); therefore, it is not reported in the results below. In addition, because sex did not have a significant effect on any of the TL measures in this cohort, male and female results are combined in the presentation of the following results. Of the two interactions reported, only the sex × training phase interaction for training duration and distance were significant. A detailed breakdown of training metrics (including average HR) across different sexes, age groups and training phases is contained in the supplementary tables (Supplementary Tables S1, S2).

**Figure 1:**
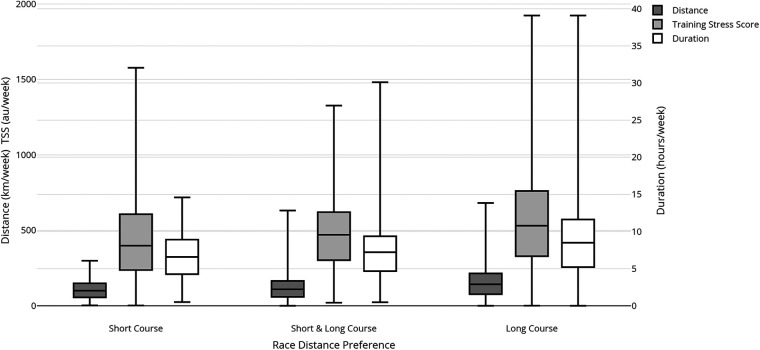
Effect on training phase on mean weekly distance (km), duration (minutes) and load (TSS).

Training phase had a relatively strong effect (with most of the TL measures (see effect sizes in Table 2; Figure 1). Total duration, total distance, and total TSS differed across training phases. TL during the Specific phase was higher (*p* < 0.001) than in the General phase, and TL in the General phase was higher (*p* < 0.001) than in the Taper/Race/Post and Off-Season phases. TL in the Off-season phase was lower (*p* < 0.05) than in all other phases.

**Figure 2:**
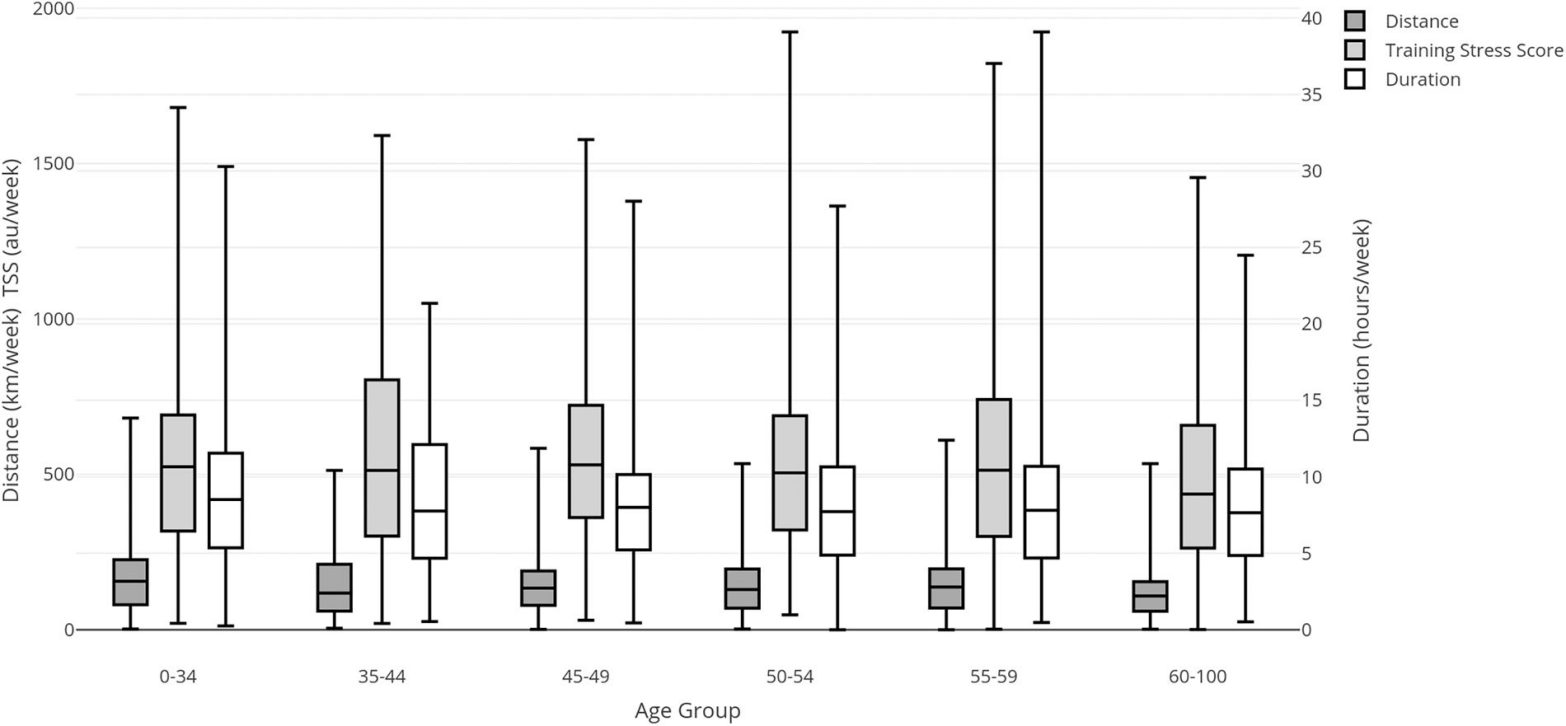
Effect of race distance preference on mean weekly distance (km), duration (minutes), and load (TSS).

Race distance preference also affected TL (see effect sizes in Table 2; Figure 2). Total duration per week, total distance per week, and total TSS per week were higher (*p* < 0.05) for triathletes who train for long-course races than for those who train for both short & long races, or for short-course races specifically.

**Figure 3:**
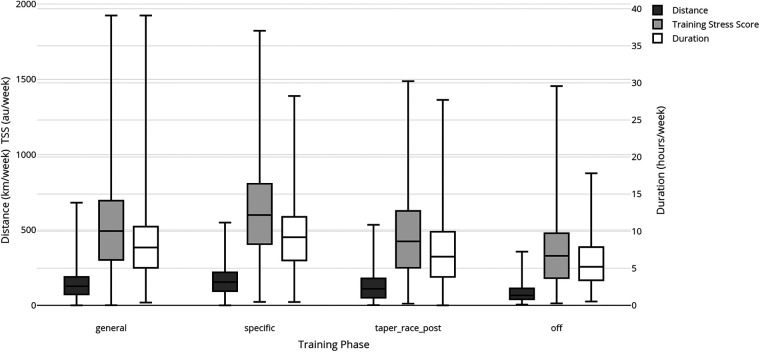
Effect of race age group on mean weekly distance (km), duration (minutes) and load (TSS).

Age group did not demonstrate an effect on TL, compared to training phase and race distance. When considered from the youngest to the oldest age group (Figure 3), measures of TL tend to increase from 0 to 34 to a peak at 35–44 and then decline progressively to the oldest age group (66–100), although none of these comparisons are statistically significant.

Of the 102 participants who started the online survey, 76 (75%) reported including strength (resistance) exercises in their training program. Typically, they performed strength training either four months of the year (38.7% of respondents) or nine months of the year (21.6%). These sessions were typically 30–50 min (mean = 40.1, SD = 17.3 min.), occurred twice per week (53.4% of respondents) and were programmed primarily in the general phase of training (84% of respondents). The average intensity (RPE) of strength sessions was 4.4 (SD = 1.3), and their training methods mainly comprised of body weight (26.1%) and free weight (26.5%) exercises.

## Discussion

This study aimed to describe a large-scale objective age-group triathlete TL dataset and evaluate whether TL varies based on sex, age, race distance preference or training phase. There were no differences in TL based on sex or age, but race distance preference and training phase were associated with differences in TL. The findings provide a descriptive age-group triathlon TL dataset on weekly duration, distance and TSS that coaches may use to help inform the design of their training programs. In addition, the results reveal how triathletes incorporate strength (resistance) training into their program and how coaches may consider athletes’ training loads based on age, race distance and training phase.

There were no differences in average HR across any levels of the four factors evaluated in this study. There is likely a difference in average HR between some types of training sessions [for example, low-intensity compared with high-intensity interval training (31)], but averaging HR across a week, as was done in the present study, would have reduced the ability to detect these differences. Nevertheless, the lack of sex- and age-related differences suggests that all triathletes in this cohort train at similar intensities relative to their maximum HR. Given that race distance and training phase were associated with the other measures of TL, based on previous research (3, 23, 32–34), we would have expected differences in HR reflecting variations in training intensity. This would have appeared as higher HR for athletes training for short-distance races and during training taper phases. The absence of a difference might reflect suboptimal training design, imperfect compliance with the prescribed training intensity, or an issue with the average weekly HR measure.

There were no age-related differences in TL, which is surprising given that younger athletes (e.g., the 18–34 age group) tend to possess higher physiological capacity (35) and there are age-related declines in physiological capacity (35, 36). Although age-related declines in physiological capacity (37–39) might be expected to reduce training load (TL), age group was not associated with weekly duration, distance, or TSS in the present cohort. Descriptively (see Supplementary Table S1), median weekly duration was similar across age groups (50th percentile: 7.65–8.51 h/wk), as was median weekly TSS (50th percentile: 437–531 TSS/wk), with a non-significant tendency for TL to peak in mid-age groups and decline in the oldest group (Figure 3). Notably, the upper end of training volume remained comparable across age groups (e.g., 90th percentile duration: ∼13.7–15.2 h/wk), suggesting that some older athletes in this self-selected cohort sustained training volumes similar to younger athletes. These findings may reflect that race-distance preference and training phase explain more variance in TL than age, and/or that this cohort represents a healthy, training-consistent subgroup of older triathletes (i.e., a survivorship/self-selection effect). We did not collect data on work hours, parenting status, or available training time; therefore, explanations based on time availability should be considered hypotheses for future research.

All TL measures differed across the training phases. There was a significant interaction between sex and training phase, suggesting small differences between males and females in the change in TL across phases. The pattern of TL change indicates that peak TL periods are used to prepare athletes for an upcoming competition (40). In contrast, TL is reduced during the period immediately before a race, during training between races, and during the training phase immediately after a race. These reductions in TL may reflect deliberate attempts to achieve supercompensation (40), permit recovery (41), and maintain physiological capacity (42). This structured approach to training phases contrasts with the more variable patterns seen in Falk-Neto's study of Olympic-distance athletes (22). These phase-specific variations in the present study indicate that age-group triathletes appear to follow periodised training plans, an evidence-based approach to optimise performance and minimise the risk of overtraining (43). The observed average phase-to-phase percentage change may guide adjusting training loads as part of a periodised (44) training program design. Relative to the General phase, measures of TL were 21% higher in the Specific phase, 14% lower in the Taper/Race/Post phase and 38% lower in the Off season phase [more detailed data from the study relevant to individual athletes are provided in the Supplementary Tables which show percentiles, by age-group (S1), and training phase (S2) and through the web app for personalised comparisons to this study's cohort (https://maps-dd.shinyapps.io/Tri_Load_Normative/).

Significantly, triathletes who trained specifically for long-course races had the highest TL, reflecting these events’ relatively high endurance demands (45). Conversely, short-course athletes displayed lower TL, aligning with the specific demands of shorter events (46). This aligns with Vleck et al.'s findings (47), which also highlight differences in training loads based on race distance. However, the Vleck et al. study (23) differs in that it focuses on world-championship-level triathletes, aligning TL data with the 95th percentile of training loads observed in this study.

The majority of triathletes in this study incorporated strength training into their training, typically either for four or nine months of the year. This aspect of training is crucial, as strength training has been shown to improve triathlon performance (48, 49) and reduce injury risk (48). However, athletes who only engage in strength training for four months may lose some benefits by the time their peak competition arrives (1). Consistent strength training throughout the year could help maintain these benefits and improve overall performance (49).

This study had several limitations. Participants may not have accurate data on age, height, or weight, or may have used inaccurate values for current power and pace thresholds entered in the TMS. The participants may also have failed to calibrate their devices before training sessions (e.g., not waiting for satellite acquisition) (16) or not correctly fit their GPS sport watch, leading to motion artifacts (50). The absence of HR differences may reflect averaging artifacts rather than training deficiencies. Of the 95 triathletes that took part in the study, 18 were female. Ideally, the split between male and female participants would have been more even to more accurately determine the association between sex and TL. Future studies of this nature should actively seek to achieve a 50:50 participation ratio. Stratified recruitment was not used in the present study; therefore, some subgroup comparisons should be interpreted cautiously. The cohort is a self-selected group of users of the TrainingPeaks platform who voluntarily submitted their data. Such respondents are likely to be more motivated, technologically engaged, and training-consistent than the wider population of recreational triathletes. This introduces a selection bias that may affect the external validity of the research. However, this is an inherent feature of all human research requiring informed consent. It is neither ethically nor practically permissible to mandate participation or compel individuals to provide personal training data. As such, some degree of volunteer bias is unavoidable when conducting ethically approved research.

Additionally, the sample was also predominantly male and skewed towards long-course athletes, which limits generalisability beyond similar age-group triathlete populations. Finally, the cross-sectional design limits causal interpretations. Future research could build on these findings to explore the relationships between training loads, race performance, and health outcomes, aiming to identify whether these loads represent optimal TL.

## Conclusions

This study utilised a large-scale training session dataset from 95 triathletes over six months, with relatively high age and geographical cohort heterogeneity, thereby increasing the dataset's diversity. However, generalisations beyond similar age-group triathlete populations should be made cautiously. The results represent the first publicly available description of a large-scale age-group triathlete TL dataset derived from training devices. Importantly, our findings demonstrate how the TL of age-group triathletes varies by training phase and race distance preference. The findings are consistent with implementing training programs that are both periodised and tailored to the demands of the race distance the athletes are training for. Notably, neither the athlete's sex nor age group were significantly associated with TL in this cohort. However, sex- and age-related interpretations should be made cautiously, given the self-selected sample and some sub-groups potentially being underpowered. The cohort's imbalance in the ratio of women to men reduces statistical power to detect sex differences in training load, and therefore, the sex-related differences identified may not indicate the absence of a true difference. These insights, including the potential for athletes to incorporate or increase resistance training, can help inform coaches and athletes. Specifically, findings may inform the development of more effective training strategies that provide meaningful context for age-group athletes to compare their training loads with those of other athletes in their age group.

## Practical considerations

- Consider tailoring training to the athlete's preferred race distance. Long-course athletes may consider a higher weekly training load, while short-course athletes may benefit from lower volumes.
- In this cohort, TL did not differ significantly by age group; however, this finding should be interpreted cautiously, given the self-selected sample and the possibility of survivorship/self-selection bias.
- Coaches, athletes, and sport scientists can refer to the supplementary tables (Supplementary S1, S2) in this article to compare their own or their athletes’ TL with that of the study's cohort across age groups, training phases, and disciplines. This may provide descriptive context for similar age-group triathletes. They can also refer to a web app at https://maps-dd.shinyapps.io/Tri_Load_Normative/ to compare their training to this data.

## Data Availability

The datasets presented in this article are not readily available because data is not provided to protect the identity of the participants. Requests to access the datasets should be directed to l.wells@deakin.edu.au.
