# Supplementary material for: Training load and intensity in triathlon: objective differences between sex, age, race distance preference and training phase across a cohort of 95 age-group triathletes over six months
Source: Front Sports Act Living. 2026 Apr 30;8:1798702. doi: 10.3389/fspor.2026.1798702 (PMC13171522; doi:10.3389/fspor.2026.1798702)
Supplement: Supplementary file 1 [file Table1.docx]

**Supplementary Tables**

**Supplementary Table 1.** **Normative total weekly training** **load** **by** **age group.***Training phases and race distance preference are aggregated.*

|  |  |  | **Percentile** | | | | | | |
| --- | --- | --- | --- | --- | --- | --- | --- | --- | --- |
| **Duration** | **Sex** | **Age** | **0.05** | **0.1** | **0.25** | **0.5** | **0.75** | **0.9** | **0.95** |
|  | **Male** | **0-34** | 2.6 | 3.3 | 4.8 | 7.2 | 10.9 | 15.9 | 19.9 |
|  |  | **35-44** | 2.4 | 3.0 | 4.4 | 6.6 | 10.1 | 14.7 | 18.3 |
|  |  | **45-49** | 2.5 | 3.2 | 4.6 | 7.0 | 10.6 | 15.4 | 19.2 |
|  |  | **50-54** | 2.5 | 3.1 | 4.6 | 6.9 | 10.5 | 15.2 | 19.1 |
|  |  | **55-59** | 2.5 | 3.2 | 4.6 | 7.0 | 10.6 | 15.4 | 19.2 |
|  |  | **59+** | 2.4 | 3.0 | 4.3 | 6.6 | 10.0 | 14.5 | 18.2 |
|  |  |  |  |  |  |  |  |  |  |
|  | **Female** | **0-34** | 2.5 | 3.1 | 4.6 | 6.9 | 10.5 | 15.3 | 19.1 |
|  |  | **35-44** | 2.3 | 2.9 | 4.2 | 6.4 | 9.7 | 14.1 | 17.6 |
|  |  | **45-49** | 2.4 | 3.0 | 4.4 | 6.7 | 10.2 | 14.8 | 18.5 |
|  |  | **50-54** | 2.4 | 3.0 | 4.4 | 6.6 | 10.1 | 14.6 | 18.3 |
|  |  | **55-59** | 2.4 | 3.0 | 4.4 | 6.7 | 10.1 | 14.8 | 18.5 |
|  |  | **59+** | 2.3 | 2.9 | 4.2 | 6.3 | 9.6 | 13.9 | 17.4 |
|  |  |  |  |  |  |  |  |  |  |
| **Distance** | **Sex** | **Age** | **0.05** | **0.1** | **0.25** | **0.5** | **0.75** | **0.9** | **0.95** |
|  | **Male** | **0-34** | 29.7 | 40.7 | 68.8 | 123.5 | 221.6 | 374.9 | 513.6 |
|  |  | **35-44** | 24.1 | 33.0 | 55.8 | 100.2 | 179.7 | 304.0 | 416.5 |
|  |  | **45-49** | 27.8 | 38.1 | 64.5 | 115.7 | 207.6 | 351.2 | 481.2 |
|  |  | **50-54** | 25.6 | 35.1 | 59.4 | 106.6 | 191.2 | 323.5 | 443.1 |
|  |  | **55-59** | 26.1 | 35.8 | 60.6 | 108.7 | 195.1 | 330.1 | 452.2 |
|  |  | **59+** | 21.1 | 29.0 | 49.0 | 87.9 | 157.7 | 266.9 | 365.7 |
|  |  |  |  |  |  |  |  |  |  |
|  | **Female** | **0-34** | 26.9 | 36.9 | 62.4 | 111.9 | 200.7 | 339.6 | 465.3 |
|  |  | **35-44** | 21.8 | 29.9 | 50.6 | 90.7 | 162.8 | 275.4 | 377.3 |
|  |  | **45-49** | 25.2 | 34.5 | 58.4 | 104.8 | 188.0 | 318.2 | 435.9 |
|  |  | **50-54** | 23.2 | 31.8 | 53.8 | 96.5 | 173.2 | 293.0 | 401.5 |
|  |  | **55-59** | 23.7 | 32.5 | 54.9 | 98.5 | 176.7 | 299.0 | 409.7 |
|  |  | **59+** | 19.2 | 26.2 | 44.4 | 79.7 | 142.9 | 241.8 | 331.3 |
|  |  |  |  |  |  |  |  |  |  |
| **TSS** | **Sex** | **Age** | **0.05** | **0.1** | **0.25** | **0.5** | **0.75** | **0.9** | **0.95** |
|  | **Male** | **0-34** | 138 | 176 | 264 | 415 | 650 | 975 | 1243 |
|  |  | **35-44** | 143 | 182 | 273 | 428 | 671 | 1005 | 1281 |
|  |  | **45-49** | 160 | 204 | 305 | 479 | 751 | 1126 | 1435 |
|  |  | **50-54** | 152 | 193 | 290 | 455 | 713 | 1070 | 1363 |
|  |  | **55-59** | 145 | 185 | 278 | 436 | 683 | 1025 | 1306 |
|  |  | **59+** | 119 | 152 | 228 | 357 | 560 | 840 | 1070 |
|  |  |  |  |  |  |  |  |  |  |
|  | **Female** | **0-34** | 140 | 179 | 268 | 421 | 660 | 990 | 1261 |
|  |  | **35-44** | 145 | 185 | 277 | 434 | 681 | 1020 | 1300 |
|  |  | **45-49** | 162 | 207 | 310 | 486 | 762 | 1143 | 1457 |
|  |  | **50-54** | 154 | 196 | 294 | 462 | 724 | 1086 | 1383 |
|  |  | **55-59** | 148 | 188 | 282 | 442 | 694 | 1040 | 1325 |
|  |  | **59+** | 121 | 154 | 231 | 362 | 568 | 852 | 1086 |

**Supplementary Table 2. Normative total weekly training** **load** **by** **training phase.***Age group and race distance preference are aggregated.*

|  |  |  | **Percentile** | | | | | | |
| --- | --- | --- | --- | --- | --- | --- | --- | --- | --- |
| **Duration** | **Sex** | **Phase** | **0.05** | **0.1** | **0.25** | **0.5** | **0.75** | **0.9** | **0.95** |
|  | **Male** | **General** | 2.6 | 3.3 | 4.7 | 7.1 | 10.6 | 15.3 | 19.1 |
|  |  | **Specific** | 3.0 | 3.7 | 5.3 | 8.0 | 12.0 | 17.3 | 21.6 |
|  |  | **Taper race post** | 1.9 | 2.4 | 3.5 | 5.3 | 7.9 | 11.4 | 14.2 |
|  |  | **Off** | 1.9 | 2.4 | 3.4 | 5.1 | 7.7 | 11.1 | 13.8 |
|  |  |  |  |  |  |  |  |  |  |
|  | **Female** | **General** | 2.5 | 3.1 | 4.5 | 6.7 | 10.1 | 14.6 | 18.2 |
|  |  | **Specific** | 2.8 | 3.5 | 5.1 | 7.6 | 11.5 | 16.6 | 20.6 |
|  |  | **Taper race post** | 1.9 | 2.3 | 3.3 | 5.0 | 7.6 | 10.9 | 13.6 |
|  |  | **Off** | 1.8 | 2.3 | 3.2 | 4.9 | 7.3 | 10.6 | 13.2 |
|  |  |  |  |  |  |  |  |  |  |
| **Distance** | **Sex** | **Phase** | **0.05** | **0.1** | **0.25** | **0.5** | **0.75** | **0.9** | **0.95** |
|  | **Male** | **General** | 26.6 | 36.4 | 61.2 | 109.1 | 194.5 | 327.4 | 447.0 |
|  |  | **Specific** | 30.3 | 41.3 | 69.5 | 124.0 | 221.1 | 372.1 | 508.1 |
|  |  | **Taper race post** | 20.1 | 27.4 | 46.1 | 82.2 | 146.6 | 246.8 | 337.0 |
|  |  | **Off** | 18.6 | 25.4 | 42.8 | 76.3 | 136.1 | 229.1 | 312.8 |
|  |  |  |  |  |  |  |  |  |  |
|  | **Female** | **General** | 24.1 | 32.9 | 55.4 | 98.7 | 176.0 | 296.2 | 404.5 |
|  |  | **Specific** | 27.4 | 37.4 | 62.9 | 112.2 | 200.0 | 336.7 | 459.7 |
|  |  | **Taper race post** | 18.2 | 24.8 | 41.7 | 74.4 | 132.7 | 223.3 | 304.9 |
|  |  | **Off** | 16.9 | 23.0 | 38.7 | 69.1 | 123.2 | 207.3 | 283.1 |
|  |  |  |  |  |  |  |  |  |  |
| **TSS** | **Sex** | **Phase** | **0.05** | **0.1** | **0.25** | **0.5** | **0.75** | **0.9** | **0.95** |
|  | **Male** | **General** | 146 | 185 | 276 | 430 | 669 | 997 | 1265 |
|  |  | **Specific** | 171 | 217 | 323 | 504 | 784 | 1168 | 1483 |
|  |  | **Taper race post** | 115 | 146 | 218 | 339 | 527 | 786 | 997 |
|  |  | **Off** | 110 | 139 | 208 | 323 | 503 | 750 | 952 |
|  |  |  |  |  |  |  |  |  |  |
|  | **Female** | **General** | 144 | 183 | 273 | 425 | 662 | 986 | 1251 |
|  |  | **Specific** | 169 | 215 | 320 | 498 | 776 | 1155 | 1467 |
|  |  | **Taper race post** | 114 | 144 | 215 | 335 | 522 | 777 | 986 |
|  |  | **Off** | 109 | 138 | 205 | 320 | 498 | 741 | 941 |
